# Vanadate Impedes Adipogenesis in Mesenchymal Stem Cells Derived from Different Depots within Bone

**DOI:** 10.3389/fendo.2016.00108

**Published:** 2016-08-03

**Authors:** Frans Alexander Jacobs, Hanél Sadie-Van Gijsen, Mari van de Vyver, William Frank Ferris

**Affiliations:** ^1^Department of Medicine, Division of Endocrinology, Faculty of Medicine and Health Sciences, Stellenbosch University, Cape Town, Western Cape, South Africa

**Keywords:** mesenchymal stem cells, bone, vanadate, adipogenesis, glucocorticoids

## Abstract

Glucocorticoid-induced osteoporosis (GIO) is associated with an increase in bone marrow adiposity, which skews the differentiation of mesenchymal stem cell (MSC) progenitors away from osteoblastogenesis and toward adipogenesis. We have previously found that vanadate, a non-specific protein tyrosine phosphatase inhibitor, prevents GIO in rats, but it was unclear whether vanadate directly influenced adipogenesis in bone-derived MSCs. For the present study, we investigated the effect of vanadate on adipogenesis in primary rat MSCs derived from bone marrow (bmMSCs) and from the proximal end of the femur (pfMSCs). By passage 3 after isolation, both cell populations expressed the MSC cell surface markers CD90 and CD106, but not the hematopoietic marker CD45. However, although variable, expression of the fibroblast marker CD26 was higher in pfMSCs than in bmMSCs. Differentiation studies using osteogenic and adipogenic induction media (OM and AM, respectively) demonstrated that pfMSCs rapidly accumulated lipid droplets within 1 week of exposure to AM, while bmMSCs isolated from the same femur only formed lipid droplets after 3 weeks of AM treatment. Conversely, pfMSCs exposed to OM produced mineralized extracellular matrix (ECM) after 3 weeks, compared to 1 week for OM-treated bmMSCs. Vanadate (10 μM) added to AM resulted in a significant reduction in AM-induced intracellular lipid accumulation and expression of adipogenic gene markers (PPARγ2, aP2, adipsin) in both pfMSCs and bmMSCs. Pharmacological concentrations of glucocorticoids (1 μM) alone did not induce lipid accumulation in either bmMSCs or pfMSCs, but resulted in significant cell death in pfMSCs. Our findings demonstrate the existence of at least two fundamentally different MSC depots within the femur and highlights the presence of MSCs capable of rapid adipogenesis within the proximal femur, an area prone to osteoporotic fractures. In addition, our results suggest that the increased bone marrow adiposity observed in GIO may not be solely due to direct effect of glucocorticoids on bone-derived MSCs, and that an increase in femur lipid content may also arise from increased adipogenesis in MSCs residing outside of the bone marrow niche.

## Introduction

Glucocorticoids (GCs) are widely used to treat a variety of inflammatory disorders, but chronic GC use can reduce bone mineral density (BMD) and lead to GC-induced osteoporosis (GIO) through an increase in bone resorption and a decrease in bone formation (1, 2). The GC-induced decrease in formation may arise from a reduction in the pool of mesenchymal stem cells (MSCs) available for osteoblastic differentiation, resulting in smaller numbers of mature bone-forming osteoblasts (1, 2).

Osteoblasts arise from multipotent MSCs, which can also differentiate into other cell-types, including adipocytes and chondrocytes (3, 4), with several lines of evidence indicating an inverse reciprocal relationship between osteoblast and adipocyte differentiation (5). Several mechanisms have been identified whereby GCs may reduce the number of osteoprogenitor cells in bone. *In vitro* studies have suggested that GCs may reduce the number of MSCs destined for osteoblastogenesis by shifting the differentiation potential of MSCs preferentially toward adipogenesis (6, 7), and this is in agreement with *in vivo* observations of GC-induced increases in marrow adiposity (8). In addition, we have previously documented the anti-proliferative effects of GCs in Naïve adipose-derived MSCs and in partially differentiated osteoblasts derived from these cells (9). Evidence from *in vivo* studies in mice and *in vitro* studies on osteoblast cell-lines indicate that GCs may also directly cause apoptosis in osteoprogenitors, osteoblasts, and osteocytes (10, 11). Taken together, these findings indicate that GCs have many deleterious effects on MSCs and on cells committed to the osteoblastic lineage, and that the prevention of these effects may restore both the number of MSCs available for osteoblastic differentiation and the number of functional osteoblasts in bone.

Earlier work in animal models found that GIO could be prevented by the non-selective protein tyrosine phosphatase inhibitor sodium orthovanadate (12), although the mechanism was not described in detail. It was subsequently shown that vanadate inhibited apoptosis of cultured osteoblasts *in vitro* and of osteocytes *in vivo* (10). Vanadate has also been shown to counteract the anti-proliferative effects of GCs on Naïve MSCs and pre-osteoblasts (9), and to inhibit adipocytic differentiation in the 3T3-L1 pre-adipocyte cell line (13). Vanadate is therefore an attractive candidate for the prevention of GIO by reversing or counteracting the effects of GCs on osteoblast precursors, possibly through a variety of mechanisms.

The aim of this study was to examine the effects of supra-physiological doses of GCs and vanadate on primary bone-derived MSCs, with a view of further understanding the etiology of GIO and how vanadate ameliorates GC-induced bone disease. Bone-derived MSCs can be isolated and cultured from various parts of long bones, including bone marrow (14), compact bone (15), the periosteal layer (16), or the epiphysis of long bones (17). However, many of these studies reported subtle differences between bone marrow-derived MSCs (bmMSCs) and MSCs derived from other areas of bone, suggesting that not all MSCs populations within bone are identical and that these different populations may respond differently during disease progression and pharmacological treatments (15–17). The typical bmMSC isolation procedure utilizes the diaphysis (shaft) of the femur, thereby excluding the proximal region of the femur, which is particularly susceptible to fracture during GIO compared to other bone sites (18, 19). We have therefore isolated MSCs from the proximal region of the femur (pfMSCs) and compared these cells with bmMSCs in order to investigate whether these two osteoprogenitor populations may differ in their responses to GCs and vanadate.

## Materials and Methods

### Experimental Animals

Experiments involving animals were approved by Stellenbosch University Ethics Committee and performed in accordance with the South African Medical Research Council Guidelines on Ethics for Medical Research in compliance with the South African Animal Protection Act (Act No. 71 of 1962). Adult male Wistar rats [12 weeks old, ~250 g mass, housed at the Stellenbosch University Animal Facility and fed *ad libitum* on standard laboratory chow (Rat and Mouse Breeder Feed, Animal Specialities, Pty. Ltd., Klapmuts, South Africa)], were used for all experiments. Animals were sacrificed *via* intraperitoneal injection with 12 mg kg^−1^ sodium pentobarbitone (Eutha-naze, Bayer, South Africa) and the femora subsequently excised.

### Cell Culture

#### Isolation of MSCs

Bone marrow-derived MSCs were isolated using a protocol that was adapted from Nadri et al. (14) and Zhu et al. (15): The tendon and muscle tissue on the femoral surface were manually removed with sterile gauze. The ends of the bones were cleaved off at the greater trochanter and saved for the isolation of pfMSCs. The bone marrow cavity was then flushed with cell isolation media [Dulbecco’s Modified Eagle Medium (DMEM) (Lonza, Verviers, Belgium)] containing 1% Penicillin/Streptomycin (P/S) (Lonza) and 20% fetal bovine serum (FBS) (Biochrom, Berlin, Germany) into a 100-mm cell culture dish using a syringe. Bone marrow from both femora was pooled and subsequently incubated overnight at 37°C in 95% humidified air containing 5% CO_2_. The following day, the culture was washed once with PBS to remove any non-adherent cells and debris, and subsequently cultured in growth media (DMEM with P/S supplemented with 10% FBS). For the isolation of pfMSCs, the protocol was adapted from Zhu et al. (15). The proximal region of the femur was macerated into 1 mm^3^ fragments and digested at 37°C for 60 min in 10 ml Hanks’ Balanced Salt Solution (Lonza) containing 0.075% (w/v) collagenase I (#CLS1, Worthington, Lakewood, NJ, USA) and 1.5% bovine serum albumin (BSA). The digested proximal femur fragments were then washed five times in DMEM and seeded in a cell culture dish with cell isolation media. After 24 h, non-adherent material was washed off with PBS and the media replaced with standard growth media.

#### Subculturing and Maintenance

All cell cultures were maintained at 37°C with 5% CO_2_ and 95% humidified air. Once the MSC cultures reached approximately 80% confluence, the cells were dissociated using 0.5% trypsin solution (Lonza) and sub-cultured at a dilution of 1:4. MSC cultures were expanded to passage 3 before being used for further experiments.

#### MSC Differentiation and Cytological Staining

All constituents of the differentiation media were purchased from Sigma-Aldrich (Schnelldorf, Germany). Differentiation experiments were performed in six-well cell culture-treated dishes (Porvair, UK). For osteoblastic differentiation, the protocol was adapted from Jaiswal et al. (20). In brief, bmMSCs were cultured to confluence before being treated with osteogenic media (OM), which consisted of growth media supplemented with 50 μM ascorbic acid, 10 mM β-glycerophosphate, and 10 nM dexamethasone, with a final ethanol concentration of 0.1%. For negative vehicle controls, cells were treated with growth media containing 0.1% ethanol. OM was replaced twice a week for the duration of osteoblastic differentiation. Once osteoblastic differentiation was complete (after day 7 for bmMSCs and day 21 for pfMSCs), mineralized extracellular deposits were stained with Alizarin Red S (Amresco, USA), using a protocol adapted from Gregory et al. (21). Briefly, cells were fixed in 70% ethanol for 10 min and stained with 40 mM Alizarin Red S dissolved in water (pH adjusted to 4.1 with 5% NH_4_OH). Excess stain was removed after 1 h, and the samples were washed twice with water. PBS (1 ml) was added to each stained well before image capturing using an Olympus CKX41 microscope (CKX41, CachN 10×/0.25 PhP objective) and a Canon EOS 600D camera.

For adipocytic differentiation, the protocol was adapted from Ogawa et al. (22). MSCs at passage 3 were cultured to 2 days post-confluence before being treated with adipogenic media (AM), which consisted of growth media supplemented with 1 μM dexamethasone, 50 μM ascorbic acid, 56 μM indomethacin, 10 μM insulin, and 0.5 mM 3-isobutyl-1-methylxanthine. The AM was replaced every 2–3 days for the duration of differentiation. For concomitant vanadate treatments, a stock solution of 10 mM sodium orthovanadate was dissolved in water and boiled as per manufacturer’s instructions (Sigma-Aldrich, Schnelldorf, Germany), and aliquots were stored at −20°C. Intracellular lipid droplets were stained with filtered 0.7% (w/v) Oil Red O (Sigma-Aldrich) in 70% isopropanol for 30 min and subsequently washed three times with water before images were captured as described above (23). For quantification of staining, isopropanol was used to extract the bound Oil Red O, and the absorbance of the extracted dye was measured at 510 nm. To correct for differences in cell density, the cells were re-stained with 0.1% (w/v) Crystal Violet (CV) nuclear stain, which was extracted with 75% ethanol, and the absorbance of the extracted dye was measured at 570 nm. Oil Red O absorption values were normalized against corresponding crystal violet absorption values (ORO/CV) and expressed as the relative triglyceride content.

### Characterization of MSC Populations using Flow Cytometry

Bone marrow-derived MSCs and pfMSCs (80% confluent; passage 3) were harvested by trypsinisation and re-suspended in PBS containing 1% BSA (Sigma-Aldrich, Berlin, Germany). Cell suspensions at a concentration of 1 × 10^6^ cells per 100 μl were co-labeled with mouse anti-rat Alexa Fluor 647-conjugated CD106 (AbDSerotec #MCA4633A647T), FITC-conjugated CD90 (BD Pharmingen, # 554897), V450-conjugated CD45 (BD Horizon, # 561587), and PE-conjugated CD26 (BD Pharmingen, # 559641). Flow cytometry was performed on a BD FACS Canto II instrument using FACSDiva software. A total of 15 000 events were recorded prior to data analysis. Since a multicolor cytometric analysis was carried out, fluorescent compensation settings were established through a compensation experiment, and regions of positive and negative staining were determined through a fluorochrome minus one (FMO) experiment. An unstained control sample was used as a negative control for gating purposes, and to measure forward and side scatter. Data analysis was performed using Flow Jo Vx (Treestar, Oregon, USA) software.

### Cell Viability

Cells at passage 3 were grown to 2 days post-confluence before being treated with 1 μM dexamethasone, in the absence or presence of 10 μM vanadate, for 7 days. For crystal violet (CV) staining, samples were fixed in 70% ethanol, stained with 0.1% CV for 5 min, and washed three times with PBS before images were captured as described above (see MSC Differentiation and Cytological Staining). Crystal violet was then extracted with 75% ethanol and the absorbance measured at 570 nm. The MTT assay protocol was adapted from the MTT-based *in vitro* toxicology assay kit from Sigma-Aldrich. Cells at passage 3 were seeded into 96-well plates and treatments commenced at 2 days post-confluence. Cells were treated for 7 days, after which 10 μl of 5 mg/ml MTT stock solution was added to each well and incubated for 2 h. The color reaction was stopped by the addition of 100 μl of 10% Triton X-100 plus 0.1N HCL in isopropanol, and samples were incubated on a plate shaker until the color product was completely dissolved. The color development was quantified spectrophotometrically at 570 nm, and background absorbance at 690 nm was subtracted from each well.

### Apoptosis Detection

For the detection and quantification of apoptosis, pfMSCs were expanded to passage 3 and grown to 2 days post-confluence before being treated with 1 μM dexamethasone and/or 10 μM vanadate for 7 days. Cells were harvested by trypsinization, subjected to centrifugation at 400 × *g* for 5 min, and cell pellets were re-suspended in PBS. Apoptosis was measured according to the PE Annexin V Apoptosis Detection Kit I (BD Pharmingen, #559763). Approximately 1 × 10^6^ cells were co-stained with PE-conjugated annexin V (a marker of early apoptosis) and 7-amino-actinomycin (7-AAD: a marker for the loss of membrane integrity and cell death) and incubated in the dark at room temperature for 15 min. Flow cytometry was performed on the FACSCanto II flow cytometer with FACSDiva software, analyzing a total of 10,000 events per sample. Further data analysis was performed on Flow Jo Vx (Treestar) software where early apoptotic cells were characterized as annexin V^+^/7-AAD^−^. Gating strategy: Unstained cells were used as a negative control, whereas cells treated with 50 μg/ml cycloheximide (4, 24, 48 h) to induce apoptosis were used as a positive control for Annexin V staining (data not shown). The positive control for the nuclear stain, 7-AAD, was obtained through prolonged exposure of cells to trypsin in order to permeabilize the cell membrane and allow 7-AAD to penetrate the cells (data not shown).

### Gene Expression Analysis (qRT-PCR)

For all gene expression analyses, cells were plated into six-well plates at passage 3. Cells were prepared for RNA extraction by lysis in Qiagen (Berlin, Germany) buffer RLT and then stored at −80°C until required. Total RNA was purified using the Qiagen RNeasy Mini kit (#74106), and 1 μg total RNA was treated with 1 U of Promega (Madison, Wisconsin, USA) RQ1 RNase-free DNase, as per manufacturer’s instructions. The DNase-treated RNA samples were used as a template for cDNA synthesis, using 20-(dT) as a primer with Promega ImProm-II reverse transcriptase. Real-time semi-quantitative RT-PCR (qRT-PCR) was performed on a Rotor-Gene 6000 (Corbett Life Science) using the Quantace Sensimix No-ROX kit (Bioline, London, England). Sequences for gene-specific primers can be found in Table 1. Relative gene expression levels were calculated according to the ΔCt method (24) and normalized to the expression of the housekeeping gene ARBP (25).

**Table 1:** **Primer sequences used in qRT-PCR**.

Gene name (*symbol*)	Ref Seq no.	Primer sequences (5′-3′)	Product size (bp)
PPARγ2 (*Pparg*) (26)	NM_013124.3	F: ACTGCCTATGAGCACTTCAC	448
		R: CAATCGGATGGTTCTTCGGA	
C/EBPα (*Cebpa*) (26)	NM_012524.3	F: TGGACAAGAACAGCAACGAG	360
		R: AATCTCCTAGTCCTGGCTTG	
aP2^a^ (*Fabp4*) (27)	NM_053365.1	F: TGAAATCACCCCAGATGACAG	185
		R: CTCATGCCCTTTCATAAACT	
Adipsin^b^ (*Cfd*)	NM_001077642.1	F: CACGTGTGCGGTGGCACCCTG	475
		R: CCCCTGCAAGTGTCCCTGCGGT	
ARBP (*Rplp0*) (25)	NM_022402.2	F: AAAGGGTCCTGGCTTTGTCT	91
		R: GCAAATGCAGATGGATCG	
Fatty acid synthase^c^ (*Fasn*)	NM_017332.1	F: GGCCTGGAGTCTATCATCAA	148
		R: CTGCACTCAGGGTGTGAT	
GLUT4^c^ (*Slc2a4*)	NM_012751.1	F: CTTCCTTCTATTTGCCGTCC	190
		R: TGCCCCTCAGTCATTCTCAT	
Msx2 (*Msx2*) (28)	NM_012982.3	F: TCACCACGTCCCAGCTTCTAG	178
		R: AGCTTTTCCAGTTCCGCCTCC	
Runx2/Cbfa1 (*Runx2*) (29)	NM_001278483.1	F: GCGGACGAGGCAAGAGTT	252
		R: TTGGTGCTGAGTTCAGGGAG	
Wnt10b^b^ (*Wnt10b*)	NM_001108111.1	F: TTCCAGCCCCGCCTACGTCCG	227
		R: CAGTGGAAACGACAGTGGC	

*^a^Forward primer designed by authors*.

*^b^Primers designed by authors*.

*^c^Primers derived from Qiagen RT2 Adipogenesis PCR array*.

### Statistical Analysis

All statistical analyses were performed with GraphPad Prism version 5.01. All data were expressed as average ± SD, and were analyzed using one-way ANOVA and Bonferroni’s multiple comparison test.

## Results

### Characterization of Isolated MSC Populations

**Figure 1:**
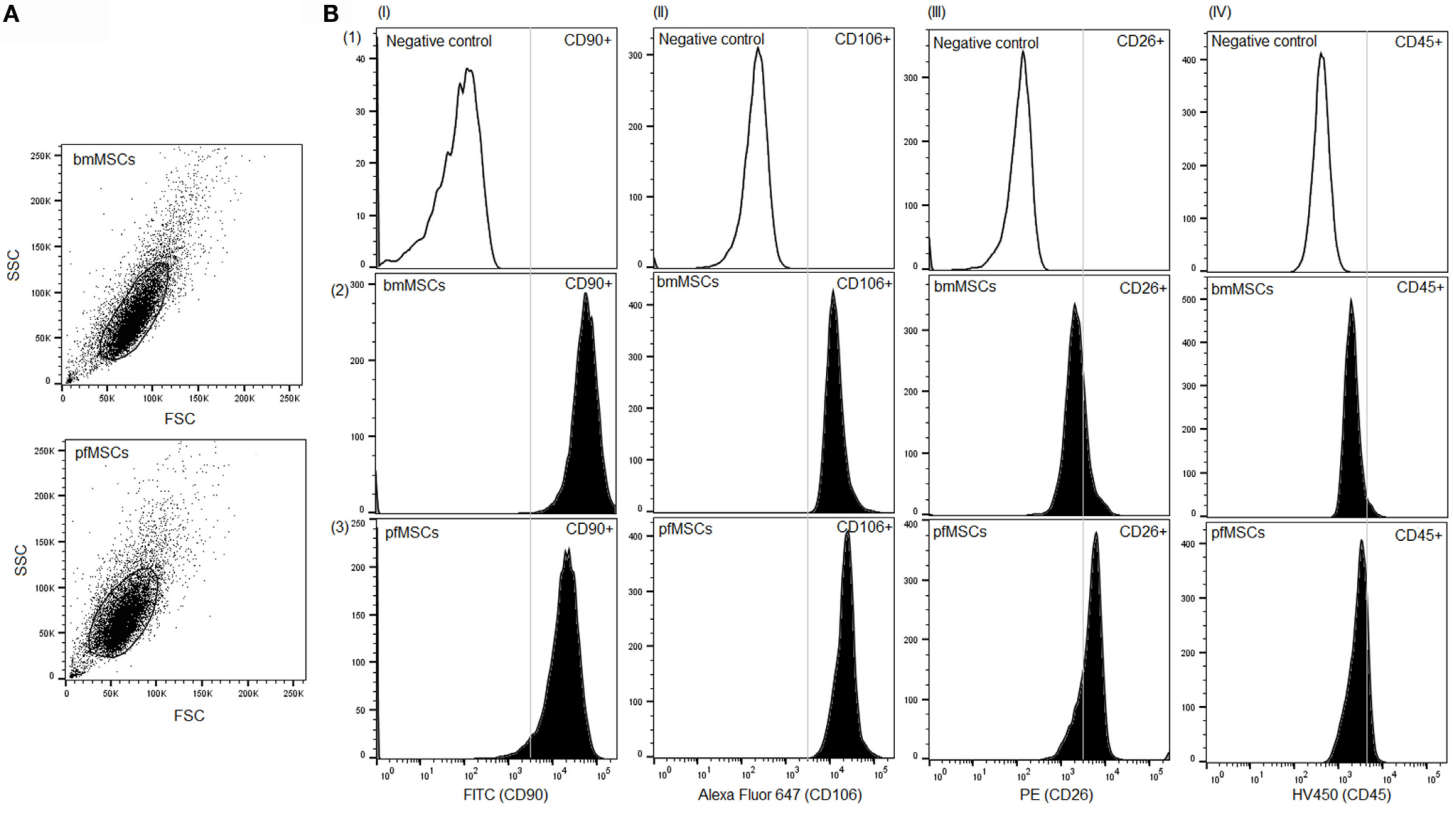
**Characterization of bone cell isolates**. **(A)** A scatter plot showing homogeneous populations of bone marrow- and proximal femur-derived cells. **(B)** Cell surface expression of the mesenchymal progenitor cell marker CD90 (column I), bmMSC marker CD106 (column II), fibroblast marker CD26 (column III), and the hematopoietic marker CD45 (column IV) was examined using flow cytometry. The first row (1) represents the negative (unstained) control samples, whereas the results for surface marker expression in bmMSCs and pfMSCs are shown in rows 2 and 3, respectively. Regions of positive staining are indicated to the right-hand side of the vertical line. Experiments were repeated in duplicate on MSCs derived from nine animals (*n* = 9), and the graphs show the results for one representative animal. For each experiment 15,000 events were recorded of which 10,000 were gated and analyzed using FlowJo Vx (Treestar) software.

The cell populations isolated from bone marrow and the proximal femur were analyzed at passage 3 using flow cytometry in order to assess the homogeneity of the populations as well as the expression of cell surface markers associated with the MSC phenotype. Both bone marrow-derived and proximal femur-derived cell cultures constituted single homogeneous populations (Figure 1A), and both cell-types expressed the MSC cell surface markers CD90 (bmMSCs 90 ± 2.5%; pfMSCs 61 ± 9%) and CD106 (bmMSCs 72 ± 4%; pfMSCs 80 ± 5%), but not the hematopoietic marker CD45 (bmMSCs 8 ± 3%; pfMSCs 12 ± 2%) (Figure 1B). Despite biological variability, the overall expression of the fibroblast marker, CD26, was slightly higher in pfMSCs (53 ± 14%) (*n* = 8) than in bmMSCs (37 ± 11%) (*n* = 9) (Figure 1B).

### Comparison of Differentiation Potential between pfMSCs and bmMSCs

**Figure 2:**
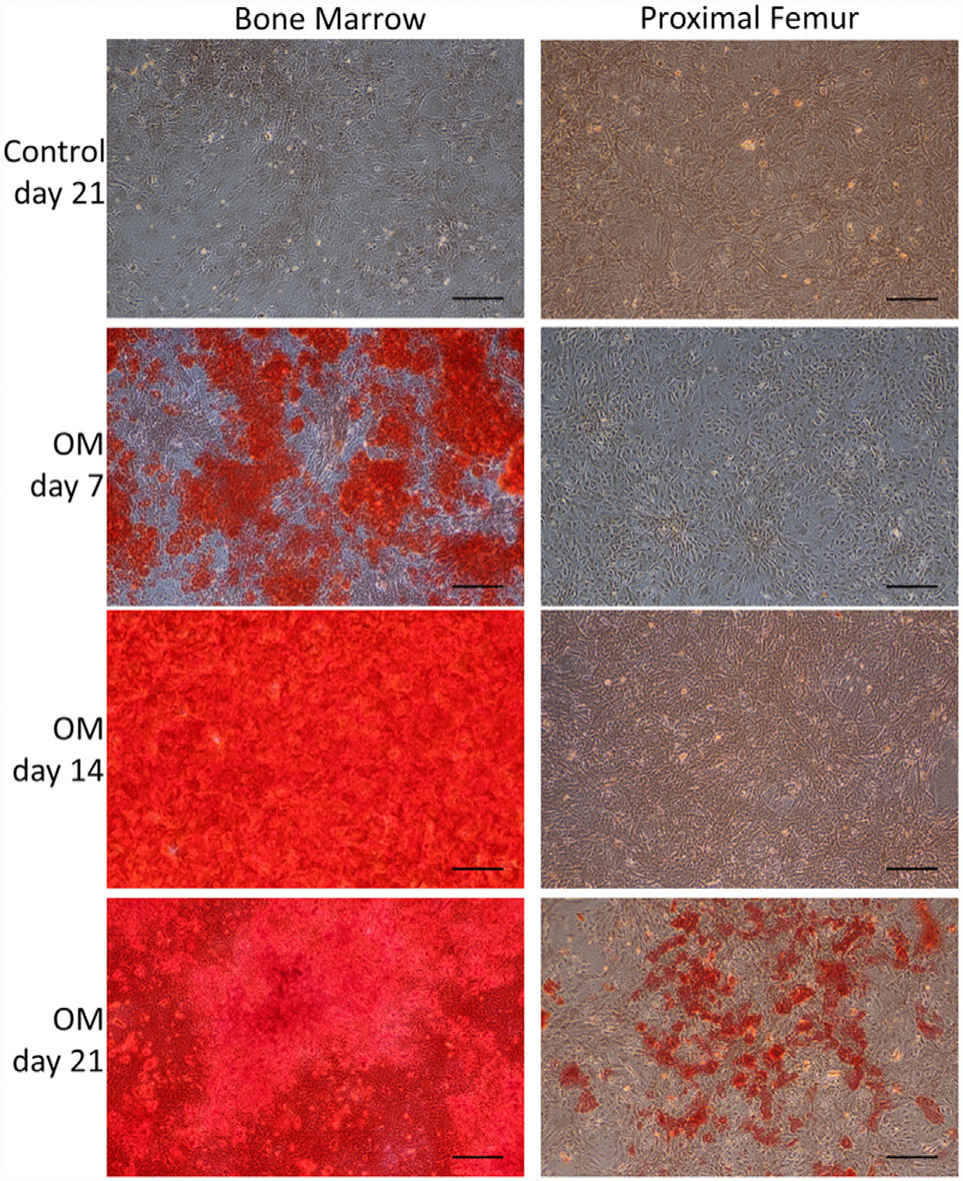
**Characterization of the osteoblastic differentiation potential of bmMSCs and pfMSCs**. Cells were treated with osteogenic media (OM) as described under Section “Materials and Methods,” for the number of days as indicated on the figure, and subsequently stained with Alizarin Red S. Images show representative results of three independent experiments, and were taken at 10× magnification, with the size bar = 1 mm.

**Figure 3:**
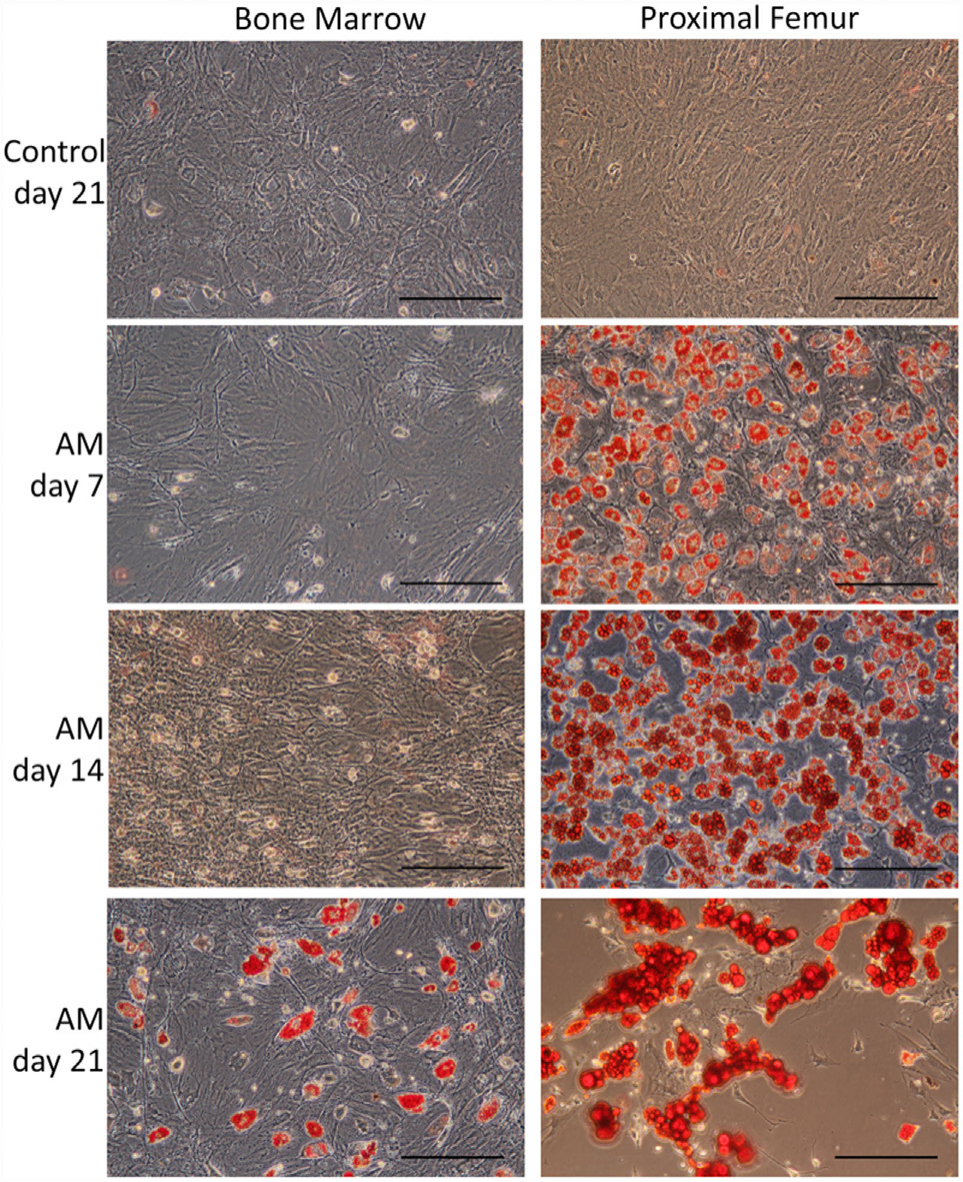
**Characterization of the adipocytic differentiation potential of bmMSCs and pfMSCs**. Cells were treated with adipogenic media (AM) as described under Section “Materials and Methods,” for the number of days as indicated on the figure, and subsequently stained with Oil Red O. Images show representative results of three independent experiments, and were taken at 20× magnification, with the size bar = 1 mm.

Bone marrow-derived MSCs and pfMSCs were treated with OM and AM to assess the osteoblastic and adipocytic differentiation potential of the cells. BmMSCs rapidly responded to OM treatment and had completely and uniformly mineralized the extracellular matrix within 7 days, while pfMSCs formed individual mineralized nodules after 21 days of OM treatment (Figure 2). In contrast, pfMSCs exhibited a strong adipogenic response, with large numbers of lipid-filled cells apparent by day 7, while bmMSCs required 21 days to form intracellular lipid droplets in a few individual cells (Figure 3).

### The Effect of Vanadate on Adipogenesis in Bone-Derived MSCs

#### Cytological Staining

**Figure 4:**
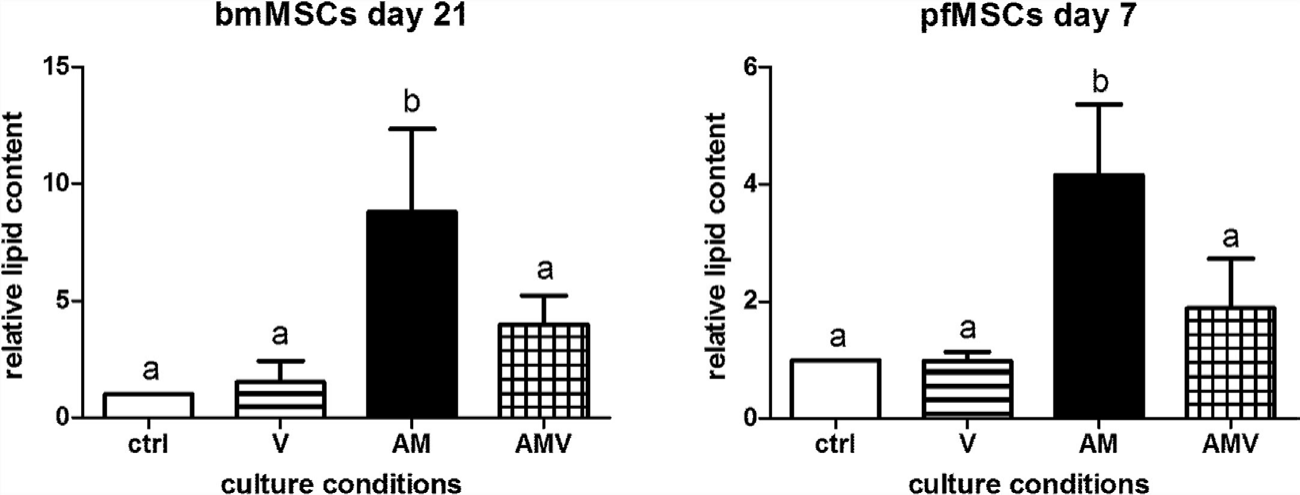
**The effect of vanadate on lipid accumulation in bmMSCs and pfMSCs**. Cells were treated with adipogenic media (AM) or AM plus 10 μM vanadate (AMV) for the number of days as indicated, before being stained with Oil Red O (ORO) and counterstained with crystal violet (CV). The ORO/CV ratio for control cells was set as 1. Different lower-case letters (a vs. b) indicates statistically significant differences (*P* < 0.05, with *n* = 4 for bmMSCs and *n* = 3 for pfMSCs).

In order to determine whether vanadate had an effect on adipogenesis in bone-derived MSCs, pfMSCs and bmMSCs were treated with AM in the absence or presence of 10 μM vanadate (treatment group AMV). Oil Red O staining was significantly decreased by approximately 50% in AMV-treated pfMSCs and bmMSCs, compared to AM-treated cells (Figure 4).

#### Gene Expression Analysis

We hypothesized that the observed partial inhibition of AM-stimulated lipid accumulation may be either due to an effect on one or more molecules in lipid sequestering or synthesizing pathways, or by globally affecting the process of adipogenesis. To ascertain whether vanadate inhibits adipogenesis, and to gain further insight into the possible mechanism(s) involved, the effect of vanadate on the expression of several adipogenesis-related genes was examined using qRT-PCR.

**Figure 5:**
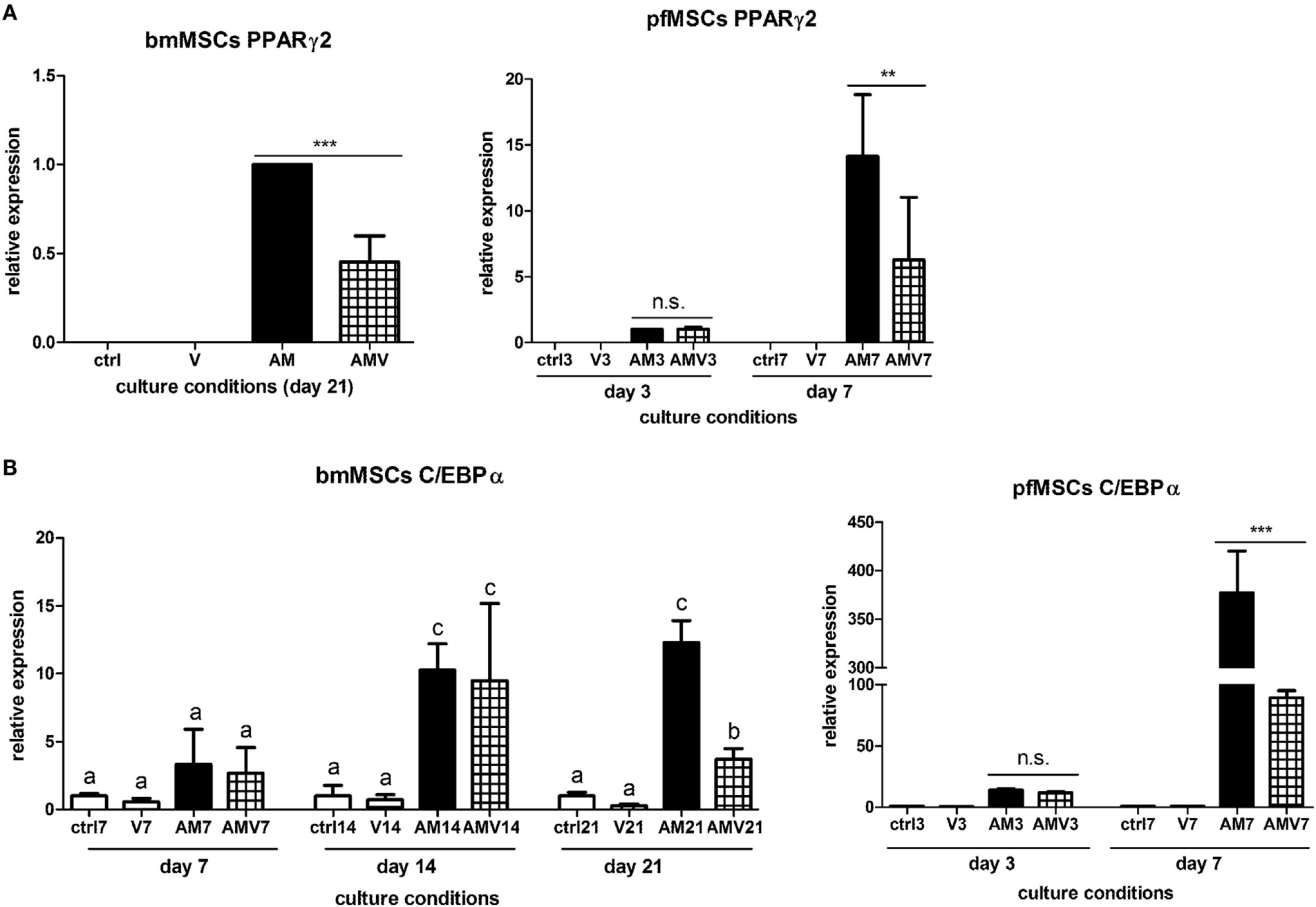
**The effect of vanadate on the expression of early markers of adipogenesis**. **(A,B)** Cells were treated with adipogenic media (AM) or AM plus 10 μM vanadate (AMV) for the number of days as indicated, and the expression of PPARγ2 **(A)** and C/EBPα **(B)** were measured by qRT-PCR. All target gene measurements were normalized to the housekeeping gene acidic ribosomal phosphoprotein (ARBP). **(A)** As PPARγ2 expression was undetectable in Naïve cells, PPARγ2 expression in day 3 AM-treated pfMSCs or day 21 AM-treated bmMSCs was set as 1. The graph presents the combined data of *n* = 3 for bmMSCs and *n* = 3 for pfMSCs. **(B)** C/EBPα expression in control cells was set as 1. The graph presents representative data from one experimental repeat out of three repeats for bmMSCs and one out of four repeats for pfMSCs.

**Figure 6:**
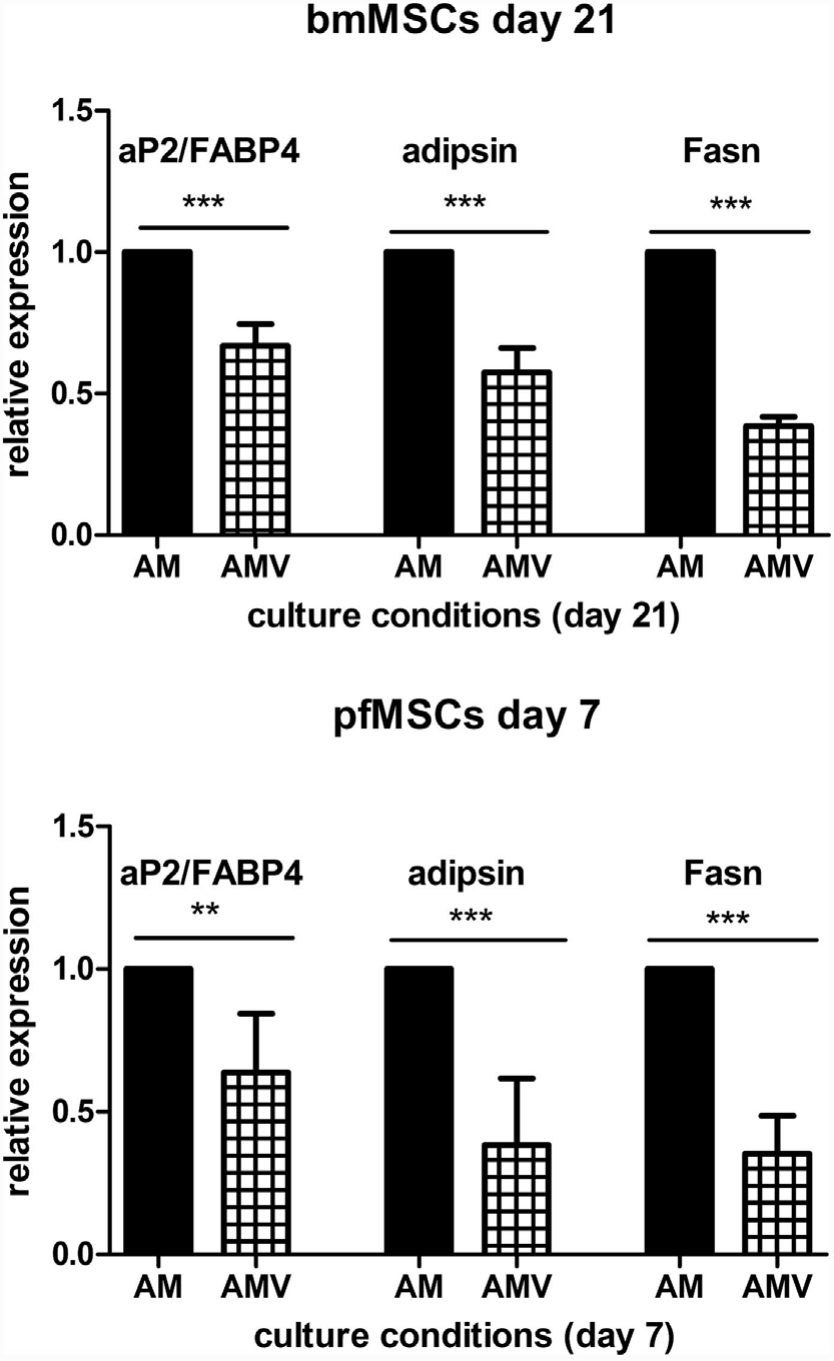
**The effect of vanadate on the expression of late markers of adipogenesis**. Cells were treated with adipogenic media (AM) or AM plus 10 μM vanadate (AMV) for the number of days as indicated, and the expression of adipocyte protein-2/fatty acid binding protein-4 (aP2/FABP4), adipsin, and fatty acid synthase (Fasn) was measured with qRT-PCR. All target gene measurements were normalized to the housekeeping gene acidic ribosomal phosphoprotein (ARBP). The expression of aP2/FABP4, adipsin, and Fasn in AM-treated cells was set as 1. The graph presents the combined data of *n* = 3 for bmMSCs and *n* = 4 for pfMSCs. Statistically significant differences are indicated as **P < 0.01 or ***P < 0.001.

The expression of the pivotal activator of adipogenesis, PPARγ2, could not be consistently observed in untreated, Naïve pfMSCs, and was completely undetectable in Naïve bmMSCs. However, PPARγ2 expression was induced in pfMSCs after 3 days of AM treatment and was maintained until day 7 (the last time-point examined for pfMSCs) (Figure 5A). Vanadate did not affect the initial upregulation of PPARγ2 expression by day 3, but reduced AM-induced PPARγ2 expression by approximately 70% by day 7 (Figure 5A). In contrast to the rapid upregulation of PPARγ2 in pfMSCs, PPARγ2 expression was only induced in bmMSCs between 14 and 21 days of AM treatment, and this induction was reduced by 30–60% by vanadate (Figure 5A). C/EBPα expression was readily detectable in untreated pfMSCs and bmMSCs (Ct values between 25 and 29), and was upregulated in response to AM at all time-points tested (Figure 5B), although the magnitude of the response to AM varied between cell isolates. In pfMSCs, C/EBPα expression exhibited a stronger induction at day 7 of AM treatment than at day 3, and a significant downregulation with vanadate at day 7 but not at day 3 (Figure 5B), similar to that observed for PPARγ2 (Figure 5A). In bmMSCs, vanadate also had very little effect on AM-induced C/EBPα expression at earlier time-points (days 7 and 14), but caused a 60–70% reduction in AM-induced C/EBPα expression by day 21 (Figure 5B). The late adipogenic markers, aP2/FABP4 (adipocyte protein-2, also known as fatty acid binding protein-4), fatty acid synthase (Fasn) and adipsin, were strongly upregulated in response to AM at day 7 in pfMSCs and day 21 in bmMSCs, although the magnitude of the responses varied dramatically. As was found for the early adipogenic markers examined, vanadate caused a significant decrease in the AM-induced expression of late adipogenic markers in both cell-types (Figure 6).

### Inherent Differences in Gene Expression Underlying the Variation in Differentiation Potential between pfMSCs and bmMSCs

**Figure 7:**
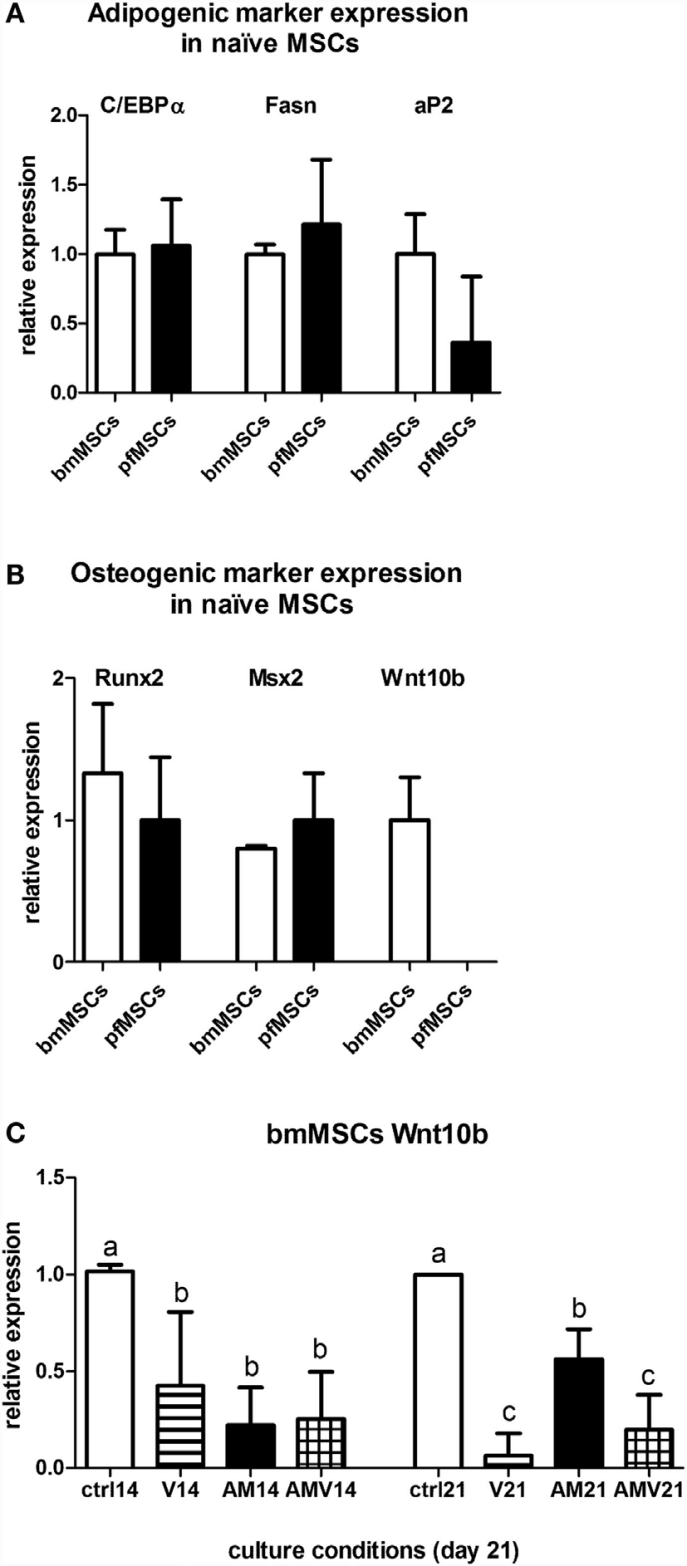
**Comparison of gene expression between bmMSCs and pfMSCs**. The relative expression levels of adipogenic genes **(A)** and osteogenic genes **(B)** in Naïve bmMSCs and pfMSCs were measured by qRT-PCR. All target gene measurements were normalized to the housekeeping gene acidic ribosomal phosphoprotein (ARBP). For each target gene, the average expression in bmMSCs (*n* = 3) was set as 1, and the expression in pfMSCs (*n* = 4) calculated relative to expression in bmMSCs. **(C)** bmMSCs were treated with adipogenic media (AM) or AM plus 10 μM vanadate (AMV) as indicated, and the expression of Wnt10b was measured by qRT-PCR after 14 and 21 days. Expression in control cells was set as 1. Different lower-case letters (a vs. b vs. c) indicates statistically significant differences (*P* < 0.05, *n* = 3).

Given the similarities in the AM-mediated upregulation of adipocyte gene expression in pfMSCs and bmMSCs, and our observations that vanadate had similar inhibitory effects on lipid accumulation and adipogenic gene expression in both cell types, we concluded that it was likely that the mechanisms governing adipogenesis and lipid accumulation were largely the same in both cell types, even though adipogenesis proceeded more rapidly in pfMSCs than in bmMSCs. However, we questioned whether differential expression of specific genes before the initiation of adipogenesis could account for the increased rate of lipid accumulation in pfMSCs, compared to bmMSCs. First, we compared the expression of adipocyte-specific genes between Naïve pfMSCs and bmMSCs using qRT-PCR. Although the expression of C/EBPα and fatty acid synthase was readily detectable in both cell types, neither was found to be differentially regulated (Figure 7A). Furthermore, the expression levels of aP2/FABP4 in Naïve cells varied considerably, but did not correlate with adipogenic potential (Figure 7A), and GLUT4 expression was found to be very low in both cell types (data not shown). It therefore appeared unlikely that the increased lipid accumulation rate of pfMSCs could be attributed to elevated adipocyte-specific gene expression in the Naïve state, and consequently the expression of anti-adipogenic genes was examined. The anti-adipogenic/pro-osteogenic factors Wnt1, Wnt3a, and sonic hedgehog (Shh) (30) were undetectable by qRT-PCR in either cell type (data not shown). Expression levels of the pro-osteogenic transcription factors Runx2 and Msx2 also did not differ between Naïve pfMSCs and bmMSCs (Figure 7B), although the anti-adipogenic/pro-osteogenic factor Wnt10b (31) was expressed in Naïve bmMSCs, but not pfMSCs (Figure 7B). Subsequently it was found that Wnt10b expression in AM-treated bmMSCs was downregulated after 14 days (Figure 7C), therefore preceding the induction of PPARγ2 expression. Wnt10b expression remained downregulated in AM-treated bmMSCs by day 21, but was unexpectedly also found to be downregulated by vanadate treatment (Figure 7C).

### The Effects of GCs on Lipid Accumulation and Viability of pfMSCs and bmMSCs

**Figure 8:**
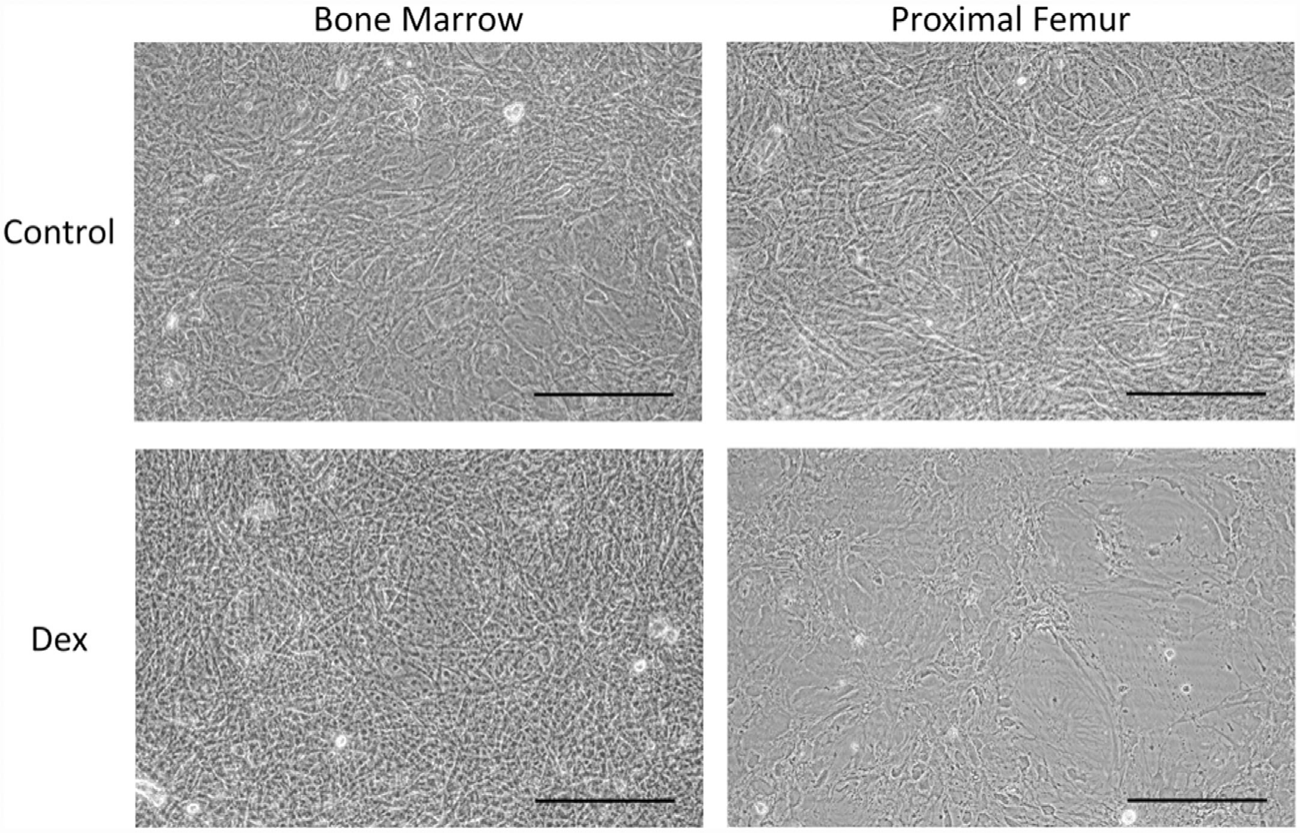
**The effect of GC treatment on bmMSCs and pfMSCs**. BmMSCs and pfMSCs were treated with 1 μM dexamethasone (Dex) for 21 days, and compared to vehicle-treated cells. Images show unstained cells at 20× magnification and are representative of three independent experiments. The size bar = 1 mm.

**Figure 9:**
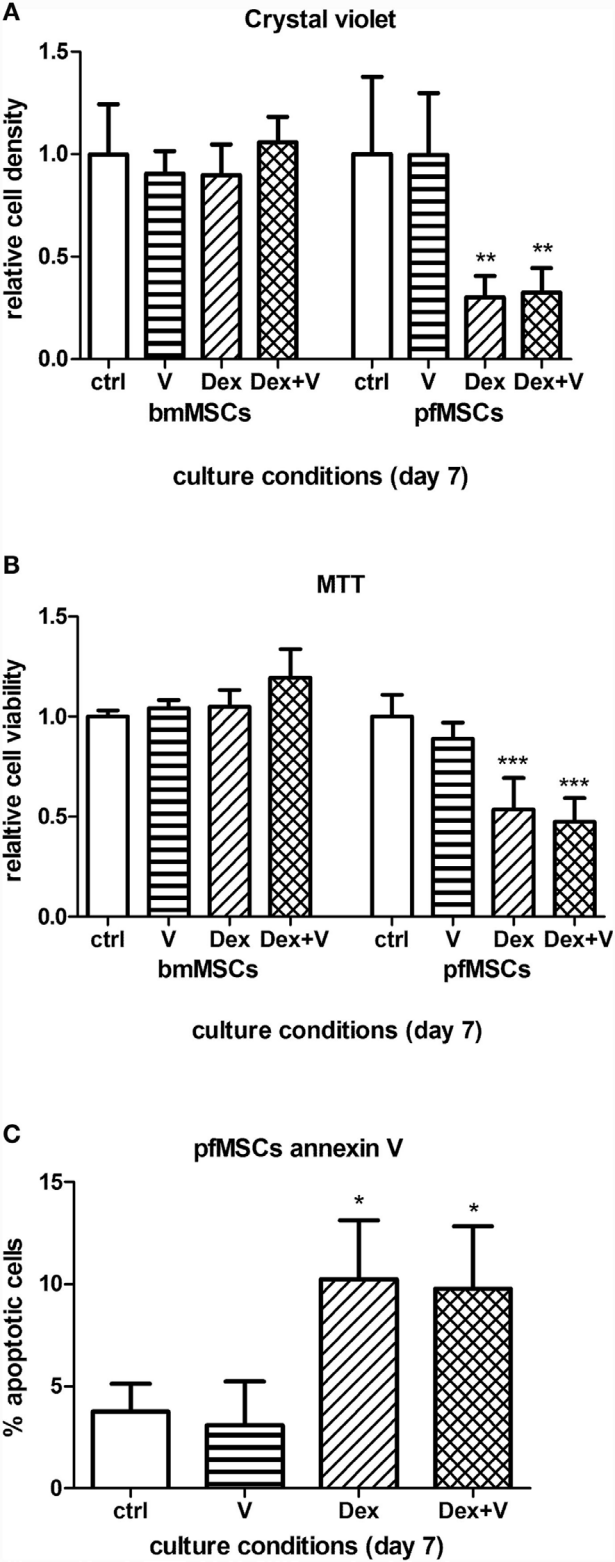
**The effects of GCs and vanadate on cell viability and apoptosis in bmMSCs and pfMSCs**. Naïve cells were treated with 1 μM dexamethasone (Dex) in the absence or presence of 10 μM vanadate. **(A,B)** Cells were stained with crystal violet **(A)** or MTT **(B)**, and the staining was quantified spectrophotometrically. Absorbance measurements of control cells were set as 1. **(C)** The percentage of apoptotic cells in treated pfMSC cultures was determined with flow cytometry, using annexin V as an apoptotic marker. Statistically significant differences are indicated as *P < 0.05, **P < 0.01, or ***P < 0.001 (*n* = 3 for bmMSCs, *n* = 3 for pfMSCs).

Given that GC-induced osteoporosis is often associated with increased bone marrow adiposity (8), it was questioned whether GCs could directly induce adipogenesis in bone-derived MSCs. PfMSCs and bmMSCs were treated with 1 μM dexamethasone (Dex) for 21 days, but no lipid accumulation was observed (Figure 8). It was also hypothesized that GCs may reduce the viability of bone-derived MSCs, as it had been previously shown that glucocorticoids induced apoptosis in bone cells *in vivo* and *in vitro* (10). In-well staining of bone-derived MSCs with crystal violet or MTT demonstrated a significant decrease in cell density of pfMSC cultures that had been treated with 1 μM Dex for 7 days (Figures 9A,B). In addition, results from the annexin V apoptosis assay indicated an increase in the percentage of apoptotic cells in Dex-treated pfMSC cultures (Figure 9C). As previous work had found that vanadate could be protective against GC-induced apoptosis in an immortalized bone cell line (10), we examined whether vanadate could counteract the apoptotic effects of Dex on pfMSCs. However, both cytological staining and apoptosis measurements showed that vanadate could not rescue pfMSCs from the negative effects of Dex treatment (Figures 9A–C). Vanadate treatment also had no effect on the viability of bmMSCs (Figures 9A,B).

## Discussion

In the present study, we report for the first time the isolation and characterization of an MSC population, residing in the proximal end of rat femurs (pfMSCs) that is phenotypically similar to, but functionally distinct from bmMSCs. Compared to bmMSCs, pfMSCs exhibited a more rapid adipogenic response but a delayed and impaired osteogenic response. However, despite these apparent differences in differentiation responses, gene expression analysis indicated that the mechanisms involved in adipogenesis and lipid accumulation are likely to be the same in both cell types. It was also found that GCs strongly reduced the viability of pfMSCs by stimulating apoptosis in these cells, while bmMSCs were resistant to the cytotoxic effects of GCs. Vanadate partially inhibited adipogenesis and lipid accumulation in both cell types, but could not reverse the GC-induced cytotoxicity in pfMSCs.

Cell surface marker analysis indicated that both pfMSCs and bmMSCs were strongly positive for the mesenchymal markers CD90 (32) and CD106 (33), and negative for the hematopoietic marker CD45 (34). However, it was found that CD26 was expressed at higher levels in pfMSCs than in bmMSCs. CD26 is considered to be a fibroblast marker, and earlier work has described that CD26 and CD106 may be used to distinguish between bmMSCs and fibroblasts (33, 35). However, fibroblasts are terminally differentiated cells, whereas the maintenance of an undifferentiated phenotype, proliferative- and bipotential differentiation capacity shown in the bmMSCS and pfMSCs in the present study indicate that these are Naïve progenitor cells, suggesting that CD26 expression is not absolutely indicative of the differentiated fibroblastic phenotype.

The most dramatic difference observed between pfMSCs and bmMSCs was in the adipocytic and osteoblastic differentiation potential, with pfMSCs being highly adipogenic and bmMSCs being highly osteogenic. Unexpectedly, despite these differences in differentiation potential, neither cell types exhibited spontaneous differentiation in culture, and we found that the expression of pro-adipogenic (PPARγ2, C/EBPα) (36) and pro-osteogenic transcription factors (Runx2, Msx2) (37, 38), as well as factors related to adipocyte function (aP2/FABP4, fatty acid synthase, GLUT4) (39, 40), did not differ between Naïve pfMSCs and bmMSCs. However, we found that Wnt10b was expressed in bmMSCs, but was absent in pfMSCs.

It is well established that an inverse relationship exists between osteoblastogenesis and adipogenesis in MSCs, with signals stimulating the one process often reciprocally inhibiting the other (5, 41). Wnt10b was first identified as a crucial “off-switch” for adipogenesis *in vitro* in 3T3-L1 pre-adipocytes (42) and subsequently *in vivo* in transgenic mice (43). Conversely, it was found that Wnt10b promoted osteoblastogenesis *in vitro* in bipotential ST2 cells (31, 44) and *in vivo* in transgenic mice (44, 45). Furthermore, proximal femur bone samples from osteoporotic women with hip fractures exhibit decreased Wnt10b expression (46), providing evidence for a relationship between Wnt10b expression, the maintenance of osteoblastic differentiation and bone strength. In addition, calvarial cultures from transgenic mice with reduced Wnt10b expression displayed dominant adipogenesis and a reduction in osteoblastogenesis (47), similar to the association between Wnt10b expression and osteoblastic differentiation seen in pfMSCs and bmMSCs in the present study. It may therefore be likely that the difference in differentiation potential between pfMSCs and bmMSCs is driven, at least in part, by a dissimilarity in Wnt10b expression. However, apart from Wnt10b expression and the concomitant delay in the onset of adipogenesis in bmMSCs, compared to pfMSCs, the profile of adipogenesis-related genes expressed in response to AM did not differ between these two cell types, indicating that the mechanism of adipocyte development from these two distinct progenitor populations was highly similar.

Glucocorticoid-induced osteoporosis is often associated with a decrease in osteoblast number and an increase in bone marrow adiposity (8). Previously, it has been found that vanadate prevented GC-induced osteoporosis *in vivo* in rats by restoring osteoblast numbers and preventing osteocyte apoptosis (10, 12). However, to our knowledge, there is no information available on the effects of vanadate on adipogenesis and lipid accumulation in bone-derived MSCs. The results presented here indicate that vanadate was able to inhibit lipid accumulation and the expression of early and late adipogenesis markers in pfMSCs and bmMSCs, demonstrating that vanadate inhibited the adipogenic program, and not just the later phases of lipid accumulation, similar to that previously found in immortalized 3T3-L1 pre-adipocytes (13). However, it should be noted that AM-induced expression of the early adipogenesis markers PPARγ2 and C/EBPα was only inhibited by vanadate at later time-points, after the expression of these genes had been upregulated by AM, suggesting the possibility that vanadate-mediated inhibition of adipogenesis involves an upstream signal that is induced during the initial phases of the adipogenic response, with consequential effects only occurring later in the differentiation process. Furthermore, we observed that the vanadate-mediated inhibition of adipogenesis in bmMSCs was not associated with upregulated expression of the anti-adipogenic Wnt10b (42, 43), but that Wnt10b was unexpectedly downregulated by AM as well as by vanadate. The inhibition of adipogenesis in MSCs by vanadate is therefore not underpinned by increased Wnt10b expression, suggesting that other signals are activated by vanadate that can inhibit adipogenesis without a requirement for Wnt10b.

Our finding that bmMSCs showed no differences in cell viability when cultured in a high concentration of Dex (1 μM) suggests that cultured primary bmMSCs are resistant to the detrimental effects of high doses of GCs, even though other studies have described decreased viability, proliferation and osteogenic potential in bmMSCs isolated from GC-treated animals (7, 48). It is therefore possible that these effects of GCs are not the result of direct GC actions at a cellular level, but that they may be mediated systemically. In contrast, pfMSCs were found to be exquisitely sensitive to the cytotoxic and apoptotic effects of GCs, but these effects could not be counteracted by vanadate co-administration. This is in contrast with previous findings, which demonstrated that *in vivo* vanadate treatment could rescue osteoblasts and osteocytes from the negative effects of GCs (10, 12), again suggesting that the effects of GCs and vanadate on bone may be systemic rather than direct.

It has been noted that GC-induced osteoporosis may result in a greater increase in fractures of the vertebrae and proximal femur, compared to fractures at other sites (18, 19), and that GC-induced fat conversion takes place in the proximal femur (8). In addition, studies on patients with GC-induced osteonecrosis of the proximal femur found a decreased proliferative capacity in isolated MSCs (49) and a decrease in progenitor cell numbers in regions adjacent to the necrotic areas (50). Moreover, it was demonstrated in rabbit models that GC treatment induced both marrow fat cell hyperplasia and hypertrophy in the proximal femur (51, 52), and that GC-induced osteonecrosis was also associated with marrow fat cell hypertrophy in this region of bone (53). While our results indicated that GCs alone could not induce lipid accumulation in either pfMSCs or bmMSCs, GC treatment reduced the viability and increased apoptosis in pfMSCs, but not in bmMSCs. These findings, taken together with the highly adipogenic nature of pfMSCs, compared to bmMSCs, suggest that distinct MSC populations may exist in the proximal femur that are more susceptible to adipogenic and apoptotic signals than other bone-derived MSC populations, such as bmMSCs. In addition, it may also be possible that the fat content of the proximal femur, which is usually labeled as “marrow adiposity,” may actually arise from distinct pfMSCs, rather than from bmMSCs from the diaphyseal region.

## Author Contributions

FJ, HS, and MV were responsible for the conceptual design of the study, experimental procedures, sample collection and analysis, interpretation of data, and preparation of the manuscript. WF contributed to the conceptual design and editing of the manuscript.

## Conflict of Interest Statement

The authors declare that the research was conducted in the absence of any commercial or financial relationships that could be construed as a potential conflict of interest.
